# Investigation of Gut Bacterial Communities of Asian Citrus Psyllid (*Diaphorina citri*) Reared on Different Host Plants

**DOI:** 10.3390/insects13080694

**Published:** 2022-08-02

**Authors:** Lixue Meng, Changxiu Xia, Zhixiong Jin, Hongyu Zhang

**Affiliations:** 1 School of Basic Medical Sciences, Hubei University of Medicine, Shiyan 442000, China; ningxueyt@126.com; 2 State Key Laboratory of Agricultural Microbiology, Key Laboratory of Horticultural Plant Biology (MOE), Institute of Urban and Horticultural Entomology, College of Plant Science and Technology, Huazhong Agricultural University, Wuhan 430070, China; 3 Ganzhou Citrus Science Institute, Ganzhou 341000, China; xia8295739@126.com; 4 The Department of Clinical Laboratory, Sinopharm Dongfeng Hospital, Hubei University of Medicine, Shiyan 442000, China; 20090569@hbmu.edu.cn

**Keywords:** *Diaphorina citri*, host species, 16S rRNA sequencing, gut microbiota

## Abstract

**Simple Summary:**

*Diaphorina citri* is a crucial natural vector of the Huanglongbing pathogen, which has devastated the citrus industry. The host plant is a critical factor that affects insect biology and its symbiont abundance. However, little is known about how host plants affect the bacterial community located in *D. citri.* In this work, the guts of five different host-plant-feeding populations (i.e., *Citrus reticulata* cv. Shatangju, *Citrus poonensis* cv. Ponkan, *Murraya paniculata* (orange jasmine), *Citrus limon* (lemon), and *Citrus sinensis* (navel orange)) were analyzed for bacterial communities by next-generation sequencing. The dominant phylum was Proteobacteria. The most common and abundant bacterial genera in *D. citri* were *Wolbachia*, *Escherichia-Shigella*, and *Candidatus Profftella*, but their relative abundance varied among the different host plant groups. There were obvious differences in the gut microbiota among the different hosts, and the gut microbe diversity was the highest in the ponkan-feeding population, while the lowest was in the Shatangju-feeding population. Overall, our findings indicate that the host plant can significantly affect the gut microbial community of *D. citri*. This result can provide new insights into the co-adaptation of *D. citri* and its symbionts.

**Abstract:**

*Diaphorina citri* Kuwayama (Hemiptera: Liviidae) can cause severe damage to citrus plants, as it transmits *Candidatus* Liberibacter spp., a causative agent of Huanglongbing disease. Symbiotic bacteria play vital roles in the ecology and biology of herbivore hosts, thereby affecting host growth and adaptation. In our research, the effects of Rutaceous plants (i.e., *Citrus reticulata* cv. Shatangju, *Citrus poonensis* cv. Ponkan, *Murraya paniculata* (orange jasmine), *Citrus limon* (lemon), and *Citrus sinensis* (navel orange)) on the gut microbiota (GM) and microbial diversity of *D. citri* adults were investigated by 16S rRNA high-throughput sequencing. It was found that Proteobacteria dominated the GM communities. The gut microbe diversity was the highest in the ponkan-feeding population, and the lowest in the Shatangju-feeding population. The NMDS analysis revealed that there were obvious differences in the GM communities among the different hosts. PICRUSt function prediction indicated significant differences in host function, and those pathways were crucial for maintaining population reproduction, growth, development, and adaptation to environmental stress in *D. citri*. Our study sheds new light on the interactions between symbionts, herbivores, and host plants and expands our knowledge on host adaptation related to GM in *D. citri*.

## 1. Introduction

The adaptation of insects to new foods and environments can be facilitated by microbial symbionts [1,2] and is critical for nutritional supplementation in insects [3,4]. Similarly, the insect gut microbiota (GM) is closely associated with plasticity, which can quickly adapt to different diets, even with alterations in the GM population structures [5]. This plasticity is crucial for insects to exploit various food sources, thus contributing to the development of host-associated differentiation, which represents an adaptive ecological strategy that reflects high species diversity in insects [6,7,8]. The characterization of GM community is crucial for revealing the ecology and biology of host insects and developing a new pest management strategy. As reviewed by Crotti et al. [9], microbes can be manipulated to enhance an SIT program, control pathogens transmitted by insects, and protect beneficial insects.

*Diaphorina citri* Kuwayama (Hemiptera: Liviidae) can cause severe damage to citrus plants, as it acts a vector of “*Candidatus* Liberibacter” (*C*Las), the causative agent related to Huanglongbing disease [10]. Excessive use of pesticides can lead to residual citrus pesticides, affect the population dynamics of natural enemies and beneficial insects, and increase the risk of environmental pollution [11]. Thus, new green control measures are of great importance for enacting control and prevention mechanisms for *D. citri*, which feeds on the phloem sap of plants in the Rutaceae family. Because of its specialized diet, *D. citri* is highly required to overcome imbalances (e.g., limited vitamins and essential amino acids) in their diet, which are commonly supplemented by microbial symbionts [12,13,14]. Thus, how does the GM communities react when *D. citri* exploits various plants as a food source? By answering this question, new environmentally friendly pest management strategy can be designed.

Insect–microbial interactions have a severe impact on natural and agricultural ecosystems. The research on insect host-related GM will provide a basic framework for further functional experiments. Despite the economic importance of *D. citri*, little is known regarding how its gut bacterial communities are influenced by host plant feeding. In this work, we used bacterial 16S-rRNA sequencing to characterize gut communities from different populations (i.e., *Citrus reticulata* cv. Shatangju, *Citrus poonensis* cv. Ponkan, *Murraya paniculata* (orange jasmine), *Citrus limon* (lemon), and *Citrus sinensis* (navel orange)). This study aimed to assess the effects of host plants on the GM communities of *D. citri* and to provide a basis for the development of efficient and green control measures.

## 2. Materials and Methods

### 2.1. Insect Sampling and Storage

Adult *D. citri* psyllids were collected from the *Citrus sinensis* (navel orange) at the Citrus Scientific Research Institute, Ganzhou, Jiangxi, and were raised on five host plant species (i.e., *Citrus reticulata* cv. Shatangju, *Citrus poonensis* cv. Ponkan, *Murraya paniculata* (orange jasmine), *Citrus limon* (lemon), and *Citrus sinensis* (navel orange)) in the institute’s net room. Regular pruning of host plant branches, fertilization, and watering ensured high numbers of tender shoots in the net. All cages and experiments were kept in a climate-controlled chamber at 27 ± 1 °C, 70–75% RH, and at 14:10 h of light:dark illumination. Adult *D. citri* of the third generation were selected as the test specimen source after 3 generations of continuous feeding to allow for the development of an adapted gut microbiota.

Each treatment (host plant) was repeated 5 times, and 200 live adult specimens were collected from each treatment. Immediately after collection, the adults were frozen at −20 °C for 5 min before dissection. The dead insects were then sterilized superficially with 70% alcohol twice for 60 s and washed twice in sterile distilled water [15]. The specimens were then placed in a phosphate-buffered solution to excise the complete gut with sterile forceps. The samples were then stored at −20 °C until DNA extraction.

### 2.2. DNA Isolation and Sequencing

The E.Z.N.ATM Soil DNA kit (Omega, Alpharetta, GA, USA) was used to isolate DNA from adult guts. The yield and quality of DNA samples were assessed by NanoDrop ND-1000 spectrophotometry (Thermo Fisher Scientific, Waltham, MA, USA) and agarose gel electrophoresis, respectively. Amplification of the 16S-rRNA V3–V4 region was conducted with 338F/806R (5′-ACT CCT ACG GGA GGC AGC A-3′ and 5′-GGA CTA CHV GGG TWT CTA AT-3′) [16]. The PCR conditions were 3 min at 95 °C, followed by 25 cycles for 30 s at 95 °C, 30 s at 55 °C, 30 s at 72 °C, and 5 min at 72 °C. All assays were performed in triplicate and then pooled to reduce PCR bias. Sequencing was conducted using an MISEQ REAGENT KIT (v2; Illumina, Inc., San Diego, CA, USA) with an Illumina MiSeq platform [17]. All data were deposited in the NCBI’s Short Read Archive (accession number: PRJNA515577). 

### 2.3. Sequence Analysis and Diversity Measures

Quantitative insights into microbial ecology (QIIME, v1.9.1) was used to process the sequencing data [18]. Paired-end sequences were aligned by Trimmomatic and FLASH, and those with >97% pairwise identity were mapped to the operational taxonomic unit (OTU) [19,20]. An open reference OTU picking strategy was used for taxonomic assignment via the Greengenes taxonomic database [21].

After rarefying the sequencing data to a depth of 20,000 reads, the microbial diversity was assessed to uncover the bacterial diversity (Shannon and Simpson) and species richness (ACE and Chao1). Beta diversity analysis was employed to assess the structural variations of microbial communities among samples based on UniFrac distance metrics [22,23] visualized via the unweighted pair-group method with arithmetic means (UPGMA), hierarchical clustering, and nonmetric multidimensional scaling (NMDS) [24]. To identify the taxa with differential abundances and indicative in each treatment, linear discriminant analysis effect size (LEfSe) analysis was conducted [25]. A logarithmic LDA score > 2 and *p* < 0.05 were deemed statistically significant. To estimate microbial functions, phylogenetic analysis of communities by reconstruction of unobserved states (PICRUSt) was conducted based on the high-quality sequences [26].

### 2.4. Statistical Analyses 

The Student’s *t*-test and one-way ANOVA followed by Tukey’s multiple comparison test were employed to compare the differences among the different treatments, and *p* < 0.05 was considered statistically significant [27]. Species abundances were determined using MetaPhlAn2 [28]. GraphPad Prism v8.0 (GraphPad Software, San Diego, CA, USA) and the R package were applied to obtain the diagrams in this study.

## 3. Results

### 3.1. High-Throughput Sequencing Data and the Diversity of GM in D. citri Populations from Various Hosts

A total of 1,111,356 sequences were derived from 25 specimens. After cleaning and trimming, 969,472 were subjected to further analysis. The length of the sequences ranged from 200 to 500 bp, with 99.93% of them having 400 to 450 bp. These sequences were further clustered into 120 bacterial OTUs. The rarefaction curves of different samples became flat, implying effective sampling and successful recovery of OTUs (Figure S1).

**Figure 1:**
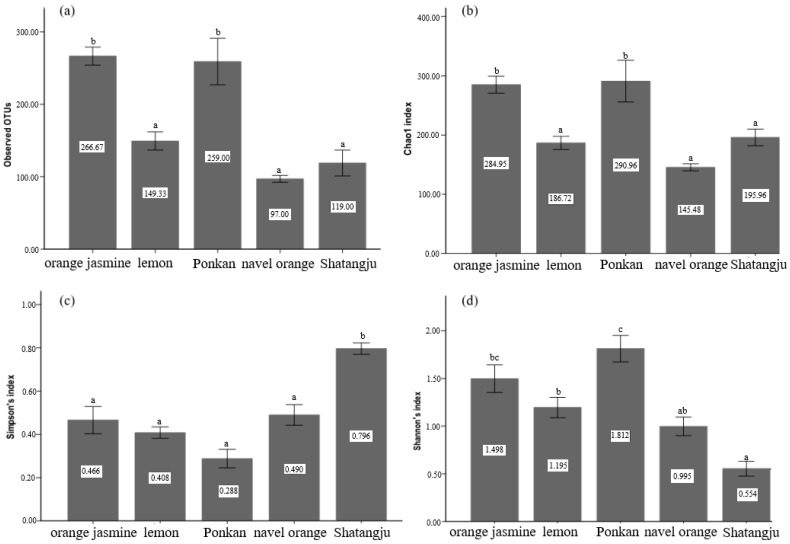
Measures of the α-diversity for each treatment: (**a**) observed OTUs; (**b**) Chao richness estimator; (**c**) Simpson’s index; (**d**) Shannon’s index. Different letters indicate that the values are significantly different (*p* < 0.01).

The alpha diversity indices were calculated to assess the bacterial diversity (Shannon and Simpson indices) and species richness (OTUs and Chao1). Analysis of species richness calculated by OTUs and Chao1 demonstrated obvious differences among the different treatments. The average species richness of the ponkan- and orange jasmine-feeding populations was significantly higher than that of the navel orange-, lemon- and Shatangju-feeding populations (*p* < 0.01) (Figure 1a,b). There were remarkable differences in the bacterial diversity calculated by the Shannon and Simpson indices. The bacterial diversities of the ponkan-feeding population were the highest as shown by the Shannon index (1.812) and Simpson index (0.288), and the lowest was found in the population feeding on Shatangju (*p* < 0.01) as shown by the Shannon index (0.554) and Simpson index (0.796) (Figure 1c,d). 

### 3.2. Comparison of GM in D. citri Populations from Various Hosts

**Figure 2:**
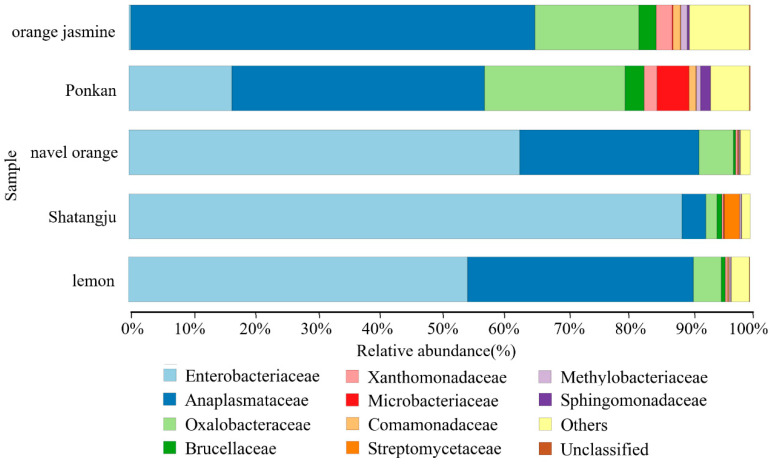
Relative abundance of families in *D. citri* gut. Only the taxa within the 10 most abundant were considered.

**Figure 3:**
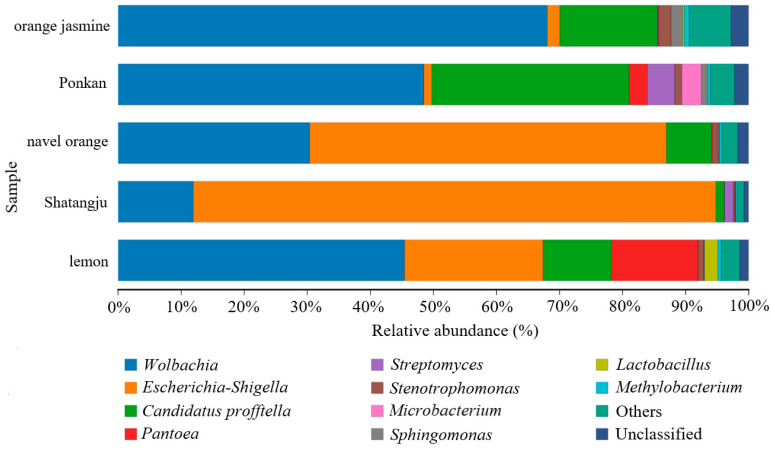
Relative abundance of genera in the gut of *D. citri*. Only the taxa within the 10 most abundant were considered.

**Figure 4:**
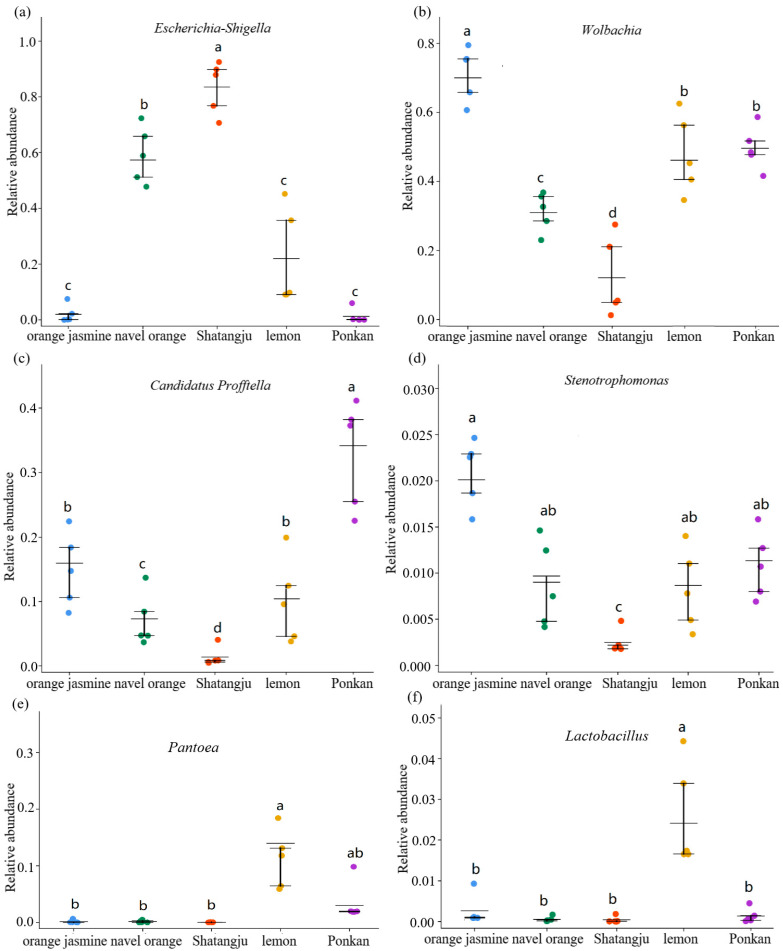
The distribution of six genera (*Escherichia-Shigella* (**a**), *Wolbachia* (**b**), *Candidatus Profftella* (**c**), *Stenotrophomonas* (**d**), *Pantoea* (**e**) and *Lactobacillus* (**f**)) differed significantly in *D. citri* gut microbes feeding on different host plants. Different letters indicate that the values are significantly different (*p* < 0.01).

The bacterial communities of all samples were dominated by Proteobacteria, but several groups contained high abundances of Actinobacteria and Firmicutes (Table S1). At the family level (Figure 2), Enterobacteriaceae was prevalent in most samples (44.69 ± 33.37%). The relative abundance of Anaplasmataceae was much higher in the orange jasmine-feeding population (65.01 ± 0.92%) than the others. A much higher relative abundance of Oxalobacteraceae in the orange jasmine- (16.73 ± 6.59%) and ponkan-feeding populations (22.69 ± 9.70%) was also observed. The most dominant bacterial genera in *D. citri* were *Wolbachia*, *Escherichia-Shigella*, and *Candidatus Profftella*, but their relative abundances varied among the different host plant groups (Figure 3, Table S2). The LSD multiple-range test showed a significantly higher relative abundance of *Escherichia-Shigella* in the Shatangju- and navel orange-feeding populations than in other host samples, especially in the orange jasmine- and ponkan-feeding populations (*p* < 0.01) (Figure 4a). The relative abundance of *Wolbachia* was significantly higher in the orange jasmine-feeding population (*p* < 0.01) (Figure 4b). *Candidatus Profftella* was present in a lower abundance in the Shatangju-feeding population but higher in the ponkan-feeding population (*p* < 0.01) (Figure 4c). Less abundant but prevalent bacteria, including *Pantoea*, *Stenotrophomonas*, *Lactobacillus*, *Microbacterium*, *Sphingomonas*, *Streptomyces*, and *Methylobacterium*, were also detected in our study (Figure 4d–f). Among them, *Microbacterium* was detected in all treatments, except in the Shatangju-feeding population. Other bacteria appeared in all treatments.

**Figure 5:**
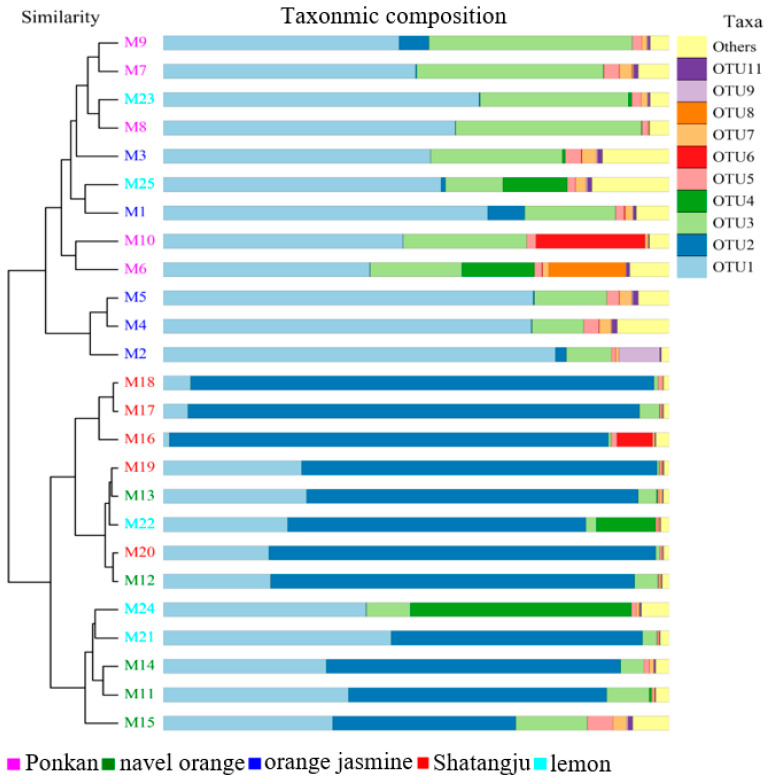
UPGMA clustering analysis of the microbiota based on weighted UniFrac distances.

**Figure 6:**
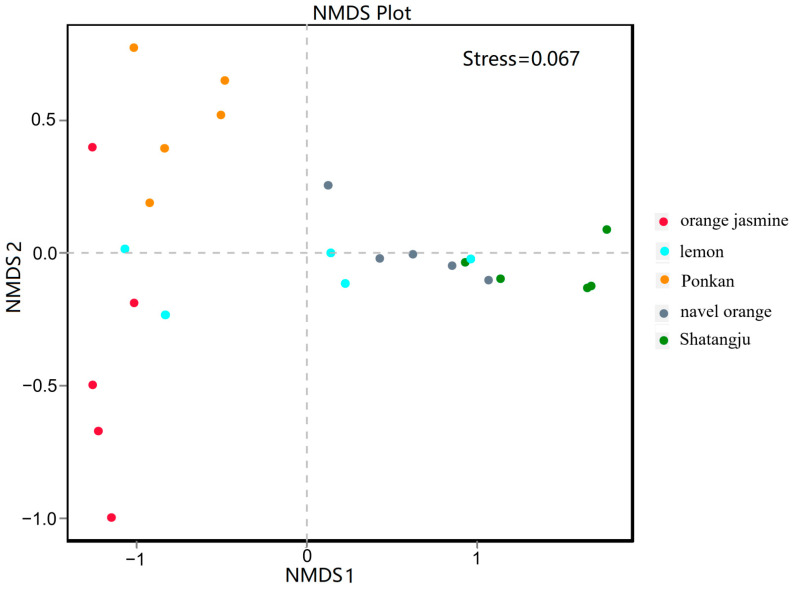
NMDS analysis of the microbial communities based on weighted UniFrac distances. Each signal represents one sample; the distance between samples demonstrates the degree of difference. Stress less than 0.2 indicates the reliability of the NMDS analysis.

The UPGMA analysis indicated that host plants affected the sample groupings. The Shatangju-feeding population samples were more characteristic of the lemon- and navel orange-feeding populations (Figure 5). The NMDS analysis of species diversity also demonstrated obvious differences among the five treatments (Figure 6). The Shatangju-feeding population, which had similar bacterial communities to the navel orange-feeding population, was separated from the orange jasmine-feeding population on NMDS1 and from the ponkan-feeding population on NMDS2 (Figure 6). While the lemon-feeding population was represented on two coordinates. The stress was 0.067, indicating that NMDS most likely reflected the degree of difference in the various samples.

LEfSe was conducted to identify specific taxa that consistently varied in abundance among the five treatments and could thus be used as biomarkers. Based on a logarithmic LDA score of 2.0 as the cutoff, a total of 14 taxa were significantly differentially represented in the five populations (Figure S2).

### 3.3. PICRUSt Analysis and Functional Prediction

**Figure 7:**
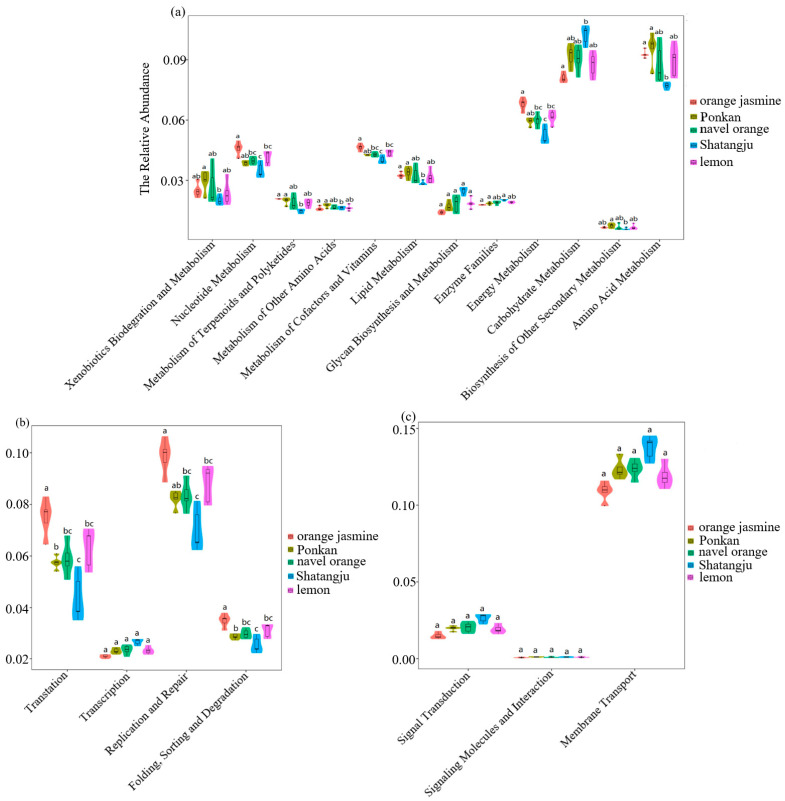
Comparison of predicted KEGG pathways of *D. citri* gut microbes feeding on different host plants. The inferred metabolic pathways are shown with Metabolism (**a**), Genetic Information Processing (**b**), Envionmental Information Processing (**c**). The bars represent the relative abundance predicted for a psyllid sample. Different letters indicate that the values are significantly different (*p* < 0.01).

To determine the effects of host plants on the GM and metabolism, PICRUSt analysis was conducted to predict the functional gene profiles of bacterial communities [26]. The KEGG pathway database was used to enrich the predicted genes. KEGG pathway analysis showed that different feeding hosts had varying “metabolism”, “genetic information processing”, and “environmental information processing” (Figure 7). The enrichment ratio of “metabolism”, which involved amino acid metabolism, energy metabolism, lipid metabolism, metabolism of polyketides and terpenoids, and xenobiotics biodegradation and metabolism, was significantly lower in the Shatangju-feeding population (*p* < 0.01). The enrichment rate for “carbohydrate metabolism” was higher in the Shatangju-feeding population (Figure 7a). Bacterial genes potentially involved in “genetic information processing” (e.g., translation, replicate and repair, folding, sorting, and degradation) were estimated to be significantly enriched in the orange jasmine-feeding population (*p* < 0.01) (Figure 7b). In “environmental information processing”, which involved three different functional groups, five populations showed differences, but these differences did not reach a significant level (Figure 7c). It can be observed that feeding on different host plants can cause varying “metabolism”, “genetic information processing”, and “environmental information processing”. These pathways are the most crucial for maintaining the population reproduction, growth, development, and adaptation to environmental stress of *D. citri*.

## 4. Discussion

Proteobacteria, Actinobacteria, Bacteroidetes, Firmicutes, and Cyanobacteria were common in all *D. citri* populations. Similar to *Grapholita molesta* [29], *Bombyx mori* [30], *Nilaparvata lugens* [31,32], mosquitoes [33,34], and *Triatoma sordida* [35], Proteobacteria had absolute dominance in *D. citri* (relative abundance > 90%). A previous study on the compositional shifts in *D. citri* microbiota through all of the life stages (i.e., egg, nymph 1–5 stages, and adult) also reported that Proteobacteria were dominant in all of the life stages of *D. citri* reared on navel orange [36]. The differences in the relative abundance of phyla provided us with a comprehensive evaluation of the differences in bacterial composition, since each phyla is usually functionally different.

Enterobacteriaceae within Proteobacteria was observed in all adult samples, and this family has been reported to play a role in sugar metabolism. Researchers have suspected that Enterobacteriaceae contributes to digestion, protection, courtship, and reproduction [37,38]. The reason this family is present in all insects may be because of its metabolic diversity, which helps insects adapt to different environments. Enterococcaceae dominated in the Shatangju-, navel orange-, and lemon-feeding populations and was significantly higher in the Shatangju-feeding population. Interestingly, our previous studies on the effects of host plants on *D. citri* development and reproduction confirmed that Shatangju was the most appropriate host for *D. citri* [39]. We can hypothesize that Enterobacteriaceae play an important role in the fitness of *D. citri*. However, additional experiments are needed to examine the roles of *D. citri* biology and its gut bacteria in the future. A higher presence of Anaplasmataceae in the ponkan- and orange jasmine-feeding populations was identified. The Anaplasmataceae was widespread in various arthropods, mainly because their versatility in helping hosts adapt to different environments.

A total of six genera that differed significantly in abundance were found in the five test populations, with *Wolbachia*, *Escherichia-Shigella*, and *Candidatus Profftella* having the most abundance. *Escherichia-Shigella*, a type of enteropathogenic genus, can cause human bacillary dysentery or Shigellosis by regulating gut tissue invasion and epithelial physiology [40]. The significance of large amounts of *Escherichia-Shigella* in the adult *D. citri* gut calls for further study. *Candidatus Profftella*, a Betaproteobacterium, is capable of producing a defensive polyketide (diaphorin) [13] and can provide essential vitamins to *D. citri* to ensure its nutritional balance. *Profftella* was reported to be localized in the bacteriome and has currently only been found in *D. citri* [41]. In the present study, *Profftella* had its highest abundance in the ponkan-feeding population, and our study is the first to report that symbiotic bacteria exist in the *D. citri* gut. *Wolbachia*, the highest relative abundance genus in the Anaplasmataceae, is systemic in *D. citri* [42,43], colocalizes in the gut [44,45], and interacts with *C*Las [45], but its functions remain unclear. In this study, *Wolbachia* had the highest abundance in the orange jasmine-feeding population, and the lowest abundance in the Shatangju-feeding population. As two dominant genera in the *D. citri* gut, it is unclear why *Profftella* and *Wolbachia* changed significantly with host plants. In addition to host plants, temperature [46], gender [46], and *C*Las-infected or not [47] could significantly affect the change of these two symbionts. *Pantoea*, *Stenotrophomonas*, *Sphingomonas*, *Methylobacterium*, *Microbacterium*, and *Lactobacillus* were classified as environmental or plant-associated microbes, which can be found during *D. citri* landing/evaluation/feeding steps on the host plants. These observed changes in the microbiome composition may serve as indicators of the ecological processes that form the host-associated microbial communities [48].

There were significant differences in GM community structures between different hosts. In the present study, NMDS revealed a distinct difference in the compositions of GM communities in *D. citri* (stress: 0.067). However, there was no significant difference between the navel orange-feeding population and the Shatangju-feeding population. One possible explanation is that Shatangju and navel orange have similar nutritional components needed for *D. citri* growth. Plant secondary metabolites and host nutrient requirements affect the composition of GM [49]. The interactions between GM and insect hosts are complex, involving morphology, behavior, physiology, and biochemistry. The microbial diversity in the Shatangju-feeding population was the lowest, suggesting Shatangju provides different nutrients for the growth and development of *D. citri*. Our previous research has shown that *D. citri* feeding on Shatangju produces more eggs than when they feed on the other four species [39]. On Ponkan, the high diversity in the GM community may contribute to the absorption of specific nutrients from unbalanced feeding and to the adaptation of *D. citri* to the feeding environment [29,50]. These results strongly suggest that the diversity and structure of the GM in *D. citri* are markedly influenced by the diet (host plants), which is in line with previous reports demonstrating that diet influences the insect GM [5,50]. Research on longhorn beetles and higher termites indicated that diet could affect the insect GM [51,52]. In mammals, diet patterns were also shown to affect the microbial community structure [53,54].

The microbial communities within the gut of a *D. citri* adult can perform many key functions, including “metabolism”, “genetic information processing”, and “environmental information processing”, that are essential to the survival of *D. citri*. Statistically significant differences among the five different feeding populations were reported for the metabolism of carbohydrate and amino acid, membrane transport, and replication and repair. We hypothesize that the differences in the function prediction could mainly be caused by the sugar, amino acid, and toxic contents as well as the secondary metabolites in the host plants. Because of the differences in nutrients and secondary metabolites, the function of the dominant GM is also different [49]. However, our results may serve as a preliminary indication of bacterial community function. Metagomeric and meta-transcriptome analysis are needed to elucidate the host–microbiome interaction and the important functions of GM so as to find new targets for controlling *D. citri*. In addition, it is necessary to compare the differences between the insect GM and host–plant microbiome. For instance, some bacteria that can be only obtained from a host plant by the insect are required to be identified, which can extend our knowledge on how certain environmental microbes are able to establish recurrent associations with hemipteran insects. In our study, the results were entirely in silico, which will require some validation work in the future due to the potential inaccuracy of high-throughput sequencing.

## 5. Conclusions

This present study conducted a detailed investigation of the GM communities present in five different host-plant-feeding populations (i.e., *Citrus reticulata* cv. Shatangju, *Citrus poonensis* cv. Ponkan, *Murraya paniculata* (orange jasmine), *Citrus limon* (lemon), and *Citrus sinensis* (navel orange)) using high-throughput sequencing technology. It was observed that host diet had a considerable influence on the formation of insect bacterial communities. Our study showed that the highest bacterial richness and diversity were in the ponkan-feeding population, and the lowest bacterial diversity were in the Shatangju-feeding population. The PICRUSt analysis showed that most functional prediction categories were related to “metabolism”, “genetic information processing”, and “environmental information processing”, which are essential for the survival of *D. citri*. Research on the GM of *D. citri* is of great significance for the development of biological control technology based on the complex relationship between vector insects and their gut bacterial communities.

## Data Availability

The data presented in this study are available upon request from the corresponding author.

## Supplementary Materials

The following supporting information can be downloaded at: https://www.mdpi.com/article/10.3390/insects13080694/s1, Figure S1: Rarefaction curve analysis of gut samples.; Figure S2: Linear Discriminant Analysis Effect Size (LEfSe) results for *D. citri* gut microbes feeding on different host plants. Table S1: The contents of main phyla in *D. citri* (%); Table S2: Mean relative abundance of the 10 most abundant genera in gut samples of *D. citri* from different host plants (%).
