# Supplementary material for: Investigation of Gut Bacterial Communities of Asian Citrus Psyllid (Diaphorina citri) Reared on Different Host Plants
Source: Insects. 2022 Aug 2;13(8):694. doi: 10.3390/insects13080694 (PMC9409139; doi:10.3390/insects13080694)
Supplement: Supplementary file 1 [file insects-13-00694-s001.zip › insects-1744794-supplementary.pdf]

insects MDPI

**Table S1.** The contents of main phyla in *D. citri* (%). Data in the table means average±SE, and means in the same row with different letters differ significantly ($p < 0.01$) according to LSD multiple range test.

| Phyla | orange jasmine | Ponkan | navel orange | Shatangju | lemon |
|---|---|---|---|---|---|
| Proteobacteria | 95.64±1.44a | 90.56±13.07a | 94.76±4.47a | 97.96±1.56a | 94.08±2.53a |
| Actinobacteria | 0.78±0.27b | 5.06±2.59a | 4.38±4.16a | 1.56±1.44b | 0.96±0.57c |
| Firmicutes | 1.06±0.50a | 0.70±0.21b | 0.54±0.25b | 0.22±0.10b | 2.42±1.46a |
| Bacteroidetes | 1.74±1.05a | 0.76±0.16 b | 0.24±0.09b | 0.18±0.04b | 0.54±0.22b |
| Cyanobacteria | 0.14±0.40a | 0.18±0.04a | 0.02±0.02b | 0.04±0.02b | 0.10±0.03ab |
| Acidobacteria | 0.22±0.14b | 1.10±0.63a | 0.00±0.00b | 0.00±0.00b | 1.52±1.40a |
| Fusobacteri | 0.04±0.02a | 0.14±0.12a | 0.00±0.00a | 0.02±0.02a | 0.00±0.00a |
| Spirochaetes | 0.08±0.08a | 0.02±0.02a | 0.02±0.02a | 0.00±0.00a | 0.04±0.04a |
| Deinococuus-Termus | 0.00±0.00a | 0.02±0.02a | 0.00±0.00a | 0.00±0.00a | 0.00±0.00a |
| Cracilibacteria | 0.00±0.00a | 0.06±0.06a | 0.00±0.00a | 0.00±0.00a | 0.00±0.00a |

**Table S2**. Mean relative abundance of the 10 most abundant genera in gut samples of *D. citri* from different host plants (%). Data in the table means average±SE, and means in the same row with different letters differ significantly ($p < 0.01$) according to LSD multiple range test.

| Genus | orange jasmine | Ponkan | navel orange | Shatangju | lemon |
|---|---|---|---|---|---|
| *Wolbachia* | 68.13±0.04a | 48.45±0.03b | 30.46±0.03c | 11.91±0.05d | 45.38±0.07b |
| *Escherichia-Shigell* | 1.96±0.01c | 1.26±0.01c | 56.49±0.06b | 82.92±0.04a | 21.97±0.13c |
| *Candidatus Profftella* | 15.45±0.03b | 31.31±0.04a | 7.14±0.02bc | 1.34±0.01c | 10.86±0.05bc |
| *Pantoea* | 0.14±0.00b | 2.92±0.03ab | 0.15±0.00b | 0.03±0.00b | 13.83±0.08a |
| *Stenotrophomonas* | 1.98±0.00a | 1.10±0.00ab | 0.93±0.00ab | 0.25±0.00c | 0.84±0.00ab |
| *Lactobacillus* | 0.25±0.00b | 0.14±0.00b | 0.05±0.00b | 0.04±0.00b | 2.04±0.03a |
| *Microbacterium* | 0.02±0.00b | 3.10±0.03a | 0.01±0.00b | 0.00±0.00b | 0.01±0.00b |
| *Sphingomonas* | 1.85±0.02a | 0.89±0.01b | 0.13±0.00b | 0.06±0.00b | 0.17±0.00b |
| *Streptomyces* | 0.08±0.00c | 4.39±0.04a | 0.01±0.00c | 1.41±0.01b | 0.02±0.00c |
| *Methylobacterium* | 0.77±0.00a | 0.44±0.00a | 0.38±0.00a | 0.27±0.00a | 0.56±0.00a |

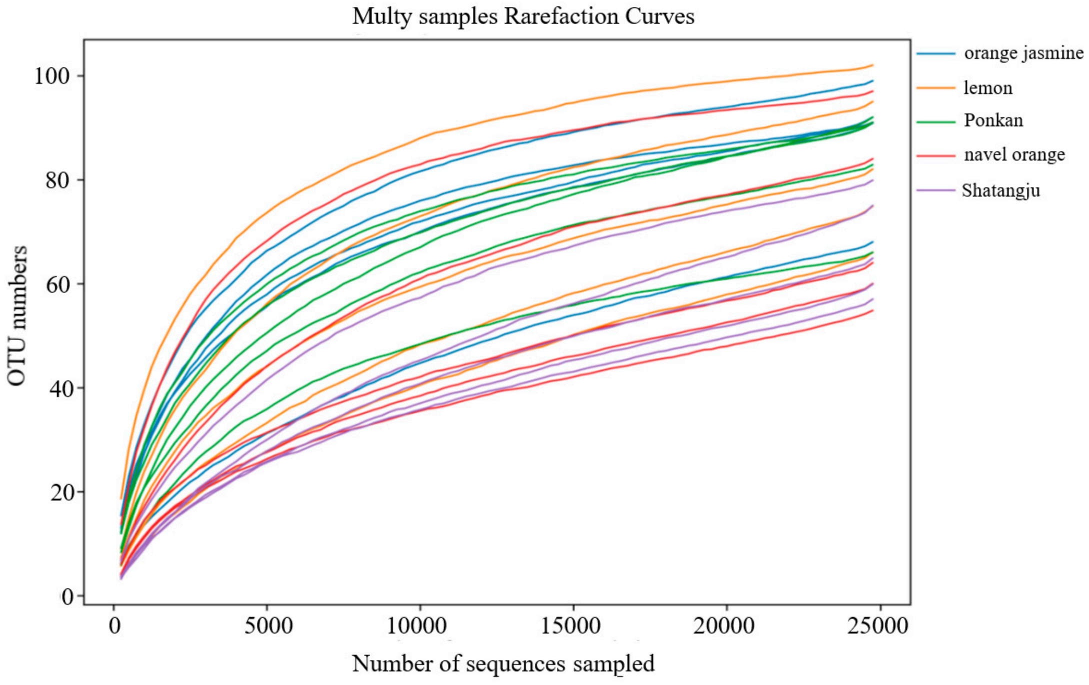


**Figure S1**. Rarefaction curve analysis of gut samples.

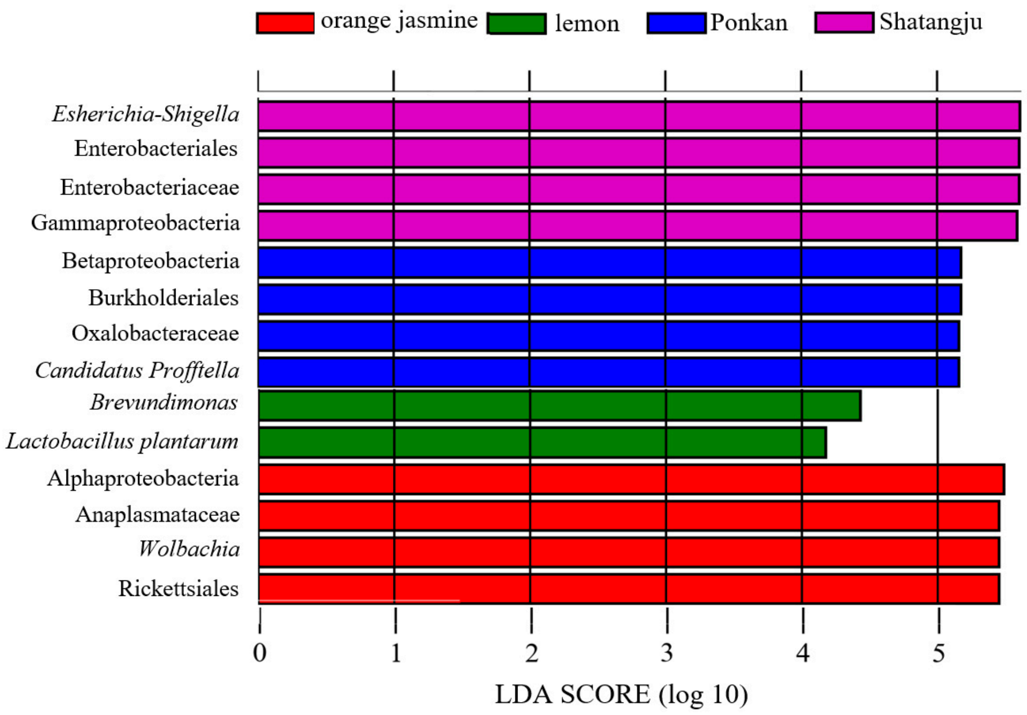


**Figure S2.** Linear Discriminant Analysis Effect Size (LEfSe) results for *D. citri* gut microbes feeding on different host plants.
